# Intracerebroventricular injection of ghrelin receptor antagonist alleviated NAFLD via improving hypothalamic insulin resistance

**DOI:** 10.22038/IJBMS.2022.64792.14272

**Published:** 2022-09

**Authors:** Yating Gong, Yaoyao Guo, Yiming Jiang, Zhiyang Xing, Heng Zhang, Hongbo Wang, Yanling Gong

**Affiliations:** 1 Department of Pharmacy, College of Chemical Engineering, Qingdao University of Science and Technology, Qingdao, China; 2 Gastrointestinal Surgery Department, Jimo District People’s Hospital, Qingdao, China

**Keywords:** Ghrelin, Hypothalamus, Insulin resistance, NAFLD, PI3K/Akt/mTOR

## Abstract

**Objective(s):**

Non-alcoholic fatty liver disease (NAFLD) is a hepatic manifestation of clinical metabolic syndrome. Insulin resistance is an important factor in the pathogenesis of NAFLD. Ghrelin, widely distributed in peripheral tissues and the central nervous system, plays a vital role in regulating food intake, energy balance, and substance metabolism. In this study, the effect of intracerebroventricular (ICV) injection of ghrelin receptor antagonist on NAFLD was explored.

**Materials and Methods:**

A rat model of NAFLD was established by feeding a high-fat diet, and a selective ghrelin receptor antagonist [D-Lys-3]-GHRP-6 was injected via ventricular intubation implantation. The serum total cholesterol (TC), triglycerides (TGs), aspartate aminotransferase (AST), alanine aminotransferase (ALT), and hepatic TGs were measured using the colorimetric method. Fasting plasma glucose (FPG) and fasting plasma insulin (FPI) were determined to calculate homeostatic model assessment insulin resistance (HOMA-IR). Hematoxylin-eosin (HE) and Oil Red O staining were conducted to observe the pathological changes and lipid accumulation in the liver. Hosphatidylinositide3-kinase (PI3K)/protein kinase B (Akt)/mammalian target of rapamycin (mTOR) signaling pathway protein expressions were measured using western blot analysis.

**Results:**

ICV injection of [D-Lys-3]-GHRP-6 significantly reduced serum lipids, transaminase, and HOMA-IR, improved liver injury, and inhibited lipid accumulation in the liver of NAFLD rats. Moreover, ICV injection of [D-Lys-3]-GHRP-6 significantly up-regulated the phosphorylation levels of PI3K/Akt/mTOR signaling protein expressions in the hypothalamus, indicating a significant improvement in hypothalamic insulin resistance.

**Conclusion:**

Blockade of central ghrelin receptor can treat NAFLD possibly via the hypothalamic PI3K/Akt/mTOR signaling pathway to improve insulin resistance.

## Introduction

Non-alcoholic fatty liver disease (NAFLD) is a hepatic manifestation of clinical metabolic syndrome and easily evolves into cirrhosis and cancer with high mortality. The most typical histological feature of NAFLD is oxidation of fat from adipose tissue into free fatty acids (FFA), which enters the liver and accumulates in hepatocytes in the form of triglycerides (1). One of the most common claims for the pathogenesis of NAFLD is the “multiple hits” doctrine. In “multiple hits”, insulin resistance is a key factor in liver steatosis (2, 3). When insulin signaling resists, FFA increases and causes liver lipid accumulation. According to a series of studies (4, 5), there are two main insulin signaling pathways: phosphatidylinositide3-kinase (PI3K)/protein kinase B (Akt/PKB) pathway and mitogenic activated protein kinase (MAPK) pathway (6). PI3K/Akt is mainly involved in glucose and lipid metabolism and related to the regulation of insulin signaling, while MAPK mainly participates in the gene regulation of insulin. The mammalian target of rapamycin (mTOR) is a downstream molecule in the PI3K/Akt signaling pathway. The phosphorylation of PI3K/Akt/mTOR activates insulin signaling. However, the inhibition of PI3K/Akt/mTOR results in increased insulin secretion and hyperinsulinemia, therefore IR takes place (7). 

Ghrelin is a peptide containing 28 amino acids and the only natural ligand of the growth hormone secretagogue receptor (GHS-R), mainly synthesized in X/A cells of the gastric mucosa (8). There are two main circulating forms for ghrelin: acylated ghrelin (AG) and non-acylated ghrelin (UAG). There are two known GHSR receptors, GHSR-1a and GHSR-1b (9). GHSR-1a is the main active type of GHSR. AG binds to GHSR-1a with its necessary acyl chain and induces feeding and obesity. GHSR-1a is widely expressed in the central and peripheral tissues. In the nervous system, GHSR-1a is mainly distributed in the hypothalamus which is involved in the regulation of substance and energy metabolism (10). There is accumulating evidence that brain insulin participates in the regulation of food intake (11). Insulin delivery to the brain regulates peripheral lipid metabolism and improves whole-body insulin sensitivity (12). The hypothalamus has been identified as an important brain region for this process (13). A nine-year follow-up study has revealed that high brain insulin sensitivity was correlated with less regain in body weight (14). Moreover, high hypothalamic insulin sensitivity was associated with less visceral fat. 

Studies have shown that long-term administration of AG can increase fasting insulin levels in rats (15, 16). After acute administration of AG in patients with a growth hormone deficiency, glucose and insulin levels rose rapidly, and AG can reduce insulin sensitivity within 6 hr (17). In our previous study, it is revealed that there was an up-regulation of hypothalamic AG and GHSR-1a expressions in the NAFLD rat induced by a high-fat diet (18). Furthermore, up-regulation of hypothalamic AG and GHSR-1a showed positive correlations with homeostatic model assessment insulin resistance (HOMA-IR) and hepatic triglycerides (TGs), respectively (18), The results suggest a potential benefit of AG blocking to NAFLD. Therefore, the effect of the central administration of a selective GHSR-1a antagonist [D-Lys-3]-GHRP-6 on NAFLD was investigated in our present study. The possible mechanism involved in hypothalamic insulin resistance was also explored. 

## Materials and Methods

***Animals and experimental design***

**Figure 1:**
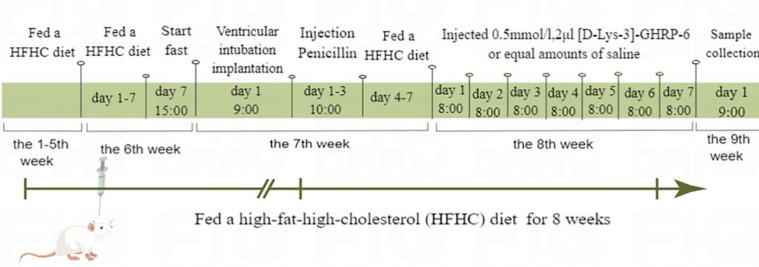
Schematic diagram of dosing time and frequency (image by Figdraw)

Thirty-two male Wistar rats (200g±20g) were purchased from Qingdao Daren Fucheng Animal Husbandry Co, LTD. All rats were fed at 22±2 ^°^C and 55±10% humidity with a 12:12-hr light-dark cycle. After one week of adaptive feeding, rats were randomly distributed into Control, NAFLD, and [D-Lys-3]-GHRP-6 groups (n=10). Rats in the NAFLD group and [D-Lys-3]-GHRP-6 group were fed a high-fat-high-cholesterol (HFHC) diet (82.5% basic diet+10% lard+2% cholesterol+0.5% bile salt+5% sucrose) for 8 weeks. Rats in the control group were fed a normal diet. At the end of the 6^th^ week, ventricular intubation implantation was performed in all rats. At the end of the 7^th^ week, rats in the administered group were given 2 μl of [D-Lys-3]-GHRP-6 (0.5mM, Absin Bioscience Inc., Shanghai, China) via ventricular intubation for intracerebroventricular injection. Rats in the control and NAFLD groups were given equal amounts of saline. [D-Lys-3]-GHRP-6 was given once a day at 8:00 a.m. for 7 days. The dosing time and frequency were shown in Figure 1. The body weight of rats was recorded during the experiment. The animal experiment was conducted according to the Guide for the Care and Use of Laboratory Animals. Two rats died one day after ventricular intubation implantation and these data were not calculated in the results. 

***Ventricular intubation implantation***

After fasting for 18 hr, rats were anesthetized using intraperitoneal injection of sodium pentobarbital (65 mg/kg). Then they were fixed on a stereotactic instrument and the skull was drilled using a dental drill. A stainless-steel guide cannula (24 G) was inserted into the rat’s third ventricle (0.86 mm posterior to bregma and 5.5 mm below the skull in the midline) (19). An acupuncture needle was placed into the cannula with a 0.5 mm length outside to prevent clogging, while taken out when delivering the drug. Penicillin was given for 3 consecutive days and the drug was delivered after a week. 

***Sample collection***

On the first day of the 9th week, rats were completely anesthetized with sodium pentobarbital (65 mg/kg) after overnight fasting. 3 ml of blood was collected via cardiac puncture immediately and serum was separated for subsequent detection. Then the rats were killed by decapitation. The brain and the liver were taken out rapidly. The hypothalamus was separated and stored at -80 ^°^C for western blot analysis. The liver was rinsed with PBS solution and weighed to calculate the liver index. The right lobe of the liver was taken and prepared with 10% liver homogenate. The left lobe of the liver was fixed in 4% paraformaldehyde, embedded in paraffin, and cut into 2-3 µm slices for hematoxylin-eosin (HE) staining. Another part of the left lobe was prepared into frozen slices for Oil Red O staining.

***Biochemical analysis***

Serum levels of total cholesterol (TC), triglycerides (TGs), aspartate aminotransferase (AST), alanine aminotransferase (ALT), and hepatic TGs were measured using commercial colorimetric kits (Nanjing Jiancheng Bioengineering Institute, Nanjing, China). Fasting plasma glucose (FPG) levels were determined using a colorimetric kit (Nanjing Jiancheng Bioengineering Institute, Nanjing, China), and fasting plasma insulin (FPI) levels were analyzed using an ELISA kit (Nanjing Jiancheng Bioengineering Institute, Nanjing, China). HOMA-IR was used to assess insulin sensitivity and was calculated using the following formula (18): HOMA-IR= (FPG×FPI)/22.5. All these biochemical procedures were performed in accordance with manufacturer instructions. 

***HE staining***

Histological morphology of the liver tissue was assessed via HE staining using paraffin-embedded slices (20, 21). The photographs were taken under a microscope (Biological microscope type XSP-2CA, Shanghai, China) under the same magnification (×100). 

***Oil Red O staining for liver***

The liver tissue was quickly frozen in liquid nitrogen, embedded with an optimal cutting temperature compound (OCT, Sakura, USA), and cut into 10 μm sections (CM1950, Leica, Germany). Oil O staining was performed using frozen slices to determine the lipid accumulation in the liver (22, 23). The photographs were taken under a microscope (Biological microscope type XSP-2CA, Shanghai, China) under the same magnification (×200). 

***Western blot analysis***

The hypothalamus tissues were lysed using RIPA (solarbio Beijing, China) containing protease inhibitors, and the protein concentration was determined using bicycin chondrogenic acid (BCA, solarbio Beijing, China) protocol (24, 25). Protein samples were denatured, after which the protein samples and marker were added to the gel sample wells and subsequently transferred to a PVDF membrane (Millipore Corporation, USA). The primary antibodies to PI3K (1:2000, Millipore Corporation, USA), Akt (1:1000, Millipore Corporation, USA), mTOR (1:1000, arigobio, Shanghai, China), p-PI3K (1:2000, CST, Boston, USA), p-Akt (1:1000, CST, Boston, USA), p-mTOR (1:1000, arigobio, Shanghai, China), β-actin (1:1000, Boster Biological Wuhan, China), and HRP labeled sheep anti-mouse secondary antibodies (1:50000, Boster Biological, Wuhan, China) Were incubated. The chemiluminescence detection was performed using an ECL reagent (Thermo Scientific, Massachusetts, America) and bands were developed with a gel imager (TANON-4600 Chemiluminescence Imager, Shanghai Tianneng Technology Co., Ltd. Shanghai, China). Specific bands were detected, analyzed, and quantified by Image J Software (v1.44, Bethesda, Rockville, MD, USA). 

***Statistical analysis***

Data were processed using SPSS17.0 software with a designed completely randomized one-way ANOVA followed by pairwise comparisons using LSD or Dunnett T3. All values are expressed as the mean±standard deviation, and *P*<0.05 indicated a statistical significance. 

## Results

***Changes in serum lipids and transaminases in rats***

**Figure 2:**
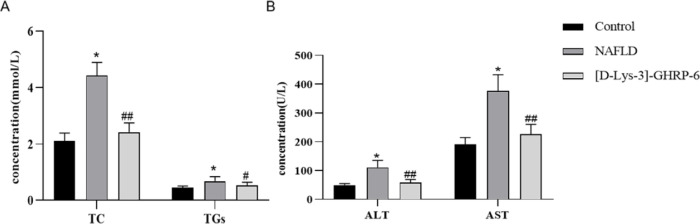
Effect of ICV injection of [D-Lys-3]-GHRP-6 on serum lipids and transaminases in NAFLD rats

After being fed an HFD diet, serum TC, TGs, ALT, and AST in the NAFLD group increased significantly when compared with the control (*P*<0.01, Figures 2A and 2B), conforming to the serological characteristics of NAFLD. However, after ICV injection of AG receptor antagonist [D-Lys-3]-GHRP-6, serum TC, TGs, ALT, and AST decreased significantly when compared with those in the NAFLD group (*P*<0.05 or 0.01, Figures 2A and 2B), indicating a lipid-lowering and hepatoprotective activities of ICV injection of [D-Lys-3]-GHRP-6.

***Changes in the liver index and hepatic TGs in rats***

**Figure 3:**
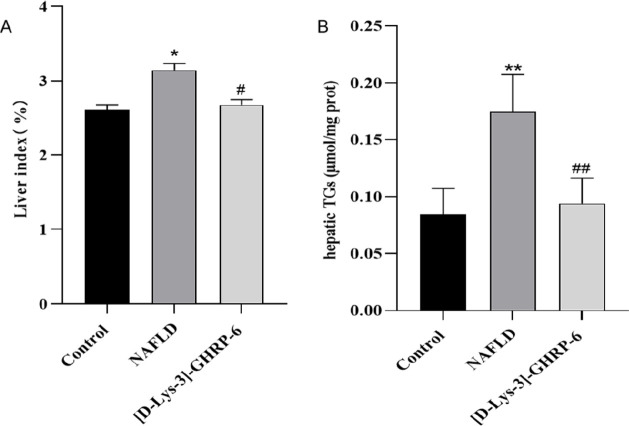
Effect of ICV injection of [D-Lys-3]-GHRP-6 on liver index and hepatic TGs in rats

As shown in Figure 3, the liver index and hepatic TGs increased in the NAFLD group (compared with the control group, *P*<0.05 or 0.01, Figures 3A and 3B) while decreasing in the [D-Lys-3]-GHRP-6 group (compared with the NAFLD group, *P*<0.05 or 0.01, Figures 3A and 3B), indicating that ICV injection of [D-Lys-3]-GHRP-6 inhibited liver hypertrophy and lipid deposition in the liver. 

***Changes in HOMA-IR in rats***

**Figure 4:**
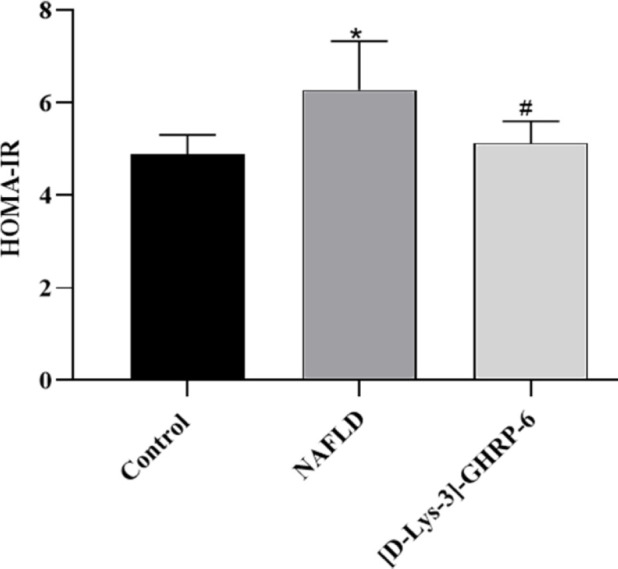
Effects of ICV injection of [D-Lys-3]-GHRP-6 on HOMA-IR in NAFLD rats

HOMA-IR in the NAFLD group was significantly higher than that in the control group (*P*<0.01, Figure 4), indicating that insulin resistance occurred in the NAFLD rats. However, ICV injection of [D-Lys-3]-GHRP-6 significantly improved insulin resistance via decreasing HOMA-IR in rats (compared with the NAFLD group, *P*<0.05, Figure 4).

***Changes in liver pathological damage in rats***

**Figure 5:**
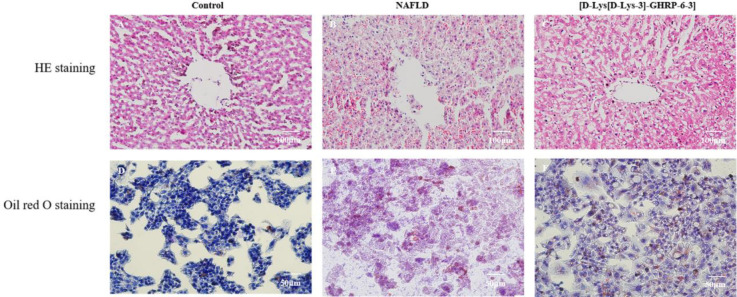
Effects of ICV injection of [D-Lys-3]-GHRP-6 on liver pathological damage in NAFLD rats. HE staining of rat liver in each group (100×): the cytoplasm is purple-red and the nucleus is blue (Figures 5A, 5B, and 5C). Oil red O staining of rat livers in each group (200×): the triglycerides were stained orange-red and the nucleus was stained blue (Figures 5D, 5E, and 5F)

ICV: Intracerebroventricular; NAFLD: Non-alcoholic fatty liver disease; HE: Hematoxylin-eosin

Liver HE staining showed the liver tissue pathological changes in rats. In the control group, the hepatic lobule structure was normal, and the hepatocytes were arranged neatly without steatosis (Figure 5A). In the NAFLD group, the hepatic lobule structure was destroyed with disordered hepatocytes. Many fat vacuoles were seen in hepatocytes, indicating steatosis occurred in rats fed an HFD diet (Figure 5B). After ICV injection of [D-Lys-3]-GHRP-6, the pathological injury improved significantly when compared with the NAFLD group, showing no significant difference with the control group (Figure 5C). 

Oil red O staining showed that there was almost no obvious lipid droplet in the hepatocytes of the control group (Figure 5D). In the NAFLD group, lipid droplets clustered and deposited in the hepatocytes (Figure 5E), in accordance with the character of hepatocytes steatosis. As expected, ICV injection of [D-Lys-3]-GHRP-6 significantly inhibited lipid accumulation in the hepatocytes induced by HFD (Figure 5F). 

***Changes in hypothalamic insulin signaling in rats***

**Figure 6:**
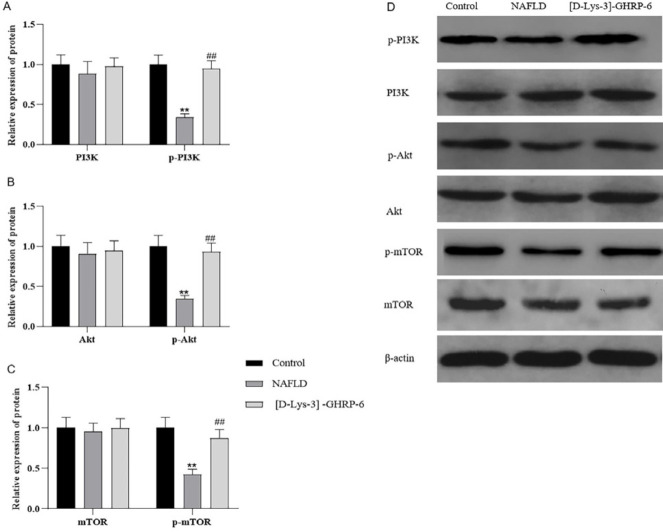
Changes of hypothalamus insulin signaling pathway protein expressions in NAFLD rats

The results of Western blot analysis showed that there was no significant difference in PI3K, Akt, and mTOR expression in the hypothalamus among control, NAFLD, and [D-Lys-3]-GHRP-6 group (n=10, *P*>0.05, Figure 6). However, the expression levels of p-PI3K, p-Akt, and p-mTOR in the hypothalamus were significantly lower in the NAFLD group when compared with the control group (n=3, *P*<0.01, Figure 6), indicating that HFD resulted in hypothalamic insulin signaling damage which was related with insulin resistance. After ICV injection of [D-Lys-3]-GHRP-6, the expression levels of p-PI3K, p-Akt, and p-mTOR in the hypothalamus increased significantly (n=10, compared with the NAFLD group, *P*<0.01, Figure 6). The results suggested that blocking the central AG activity via ghrelin receptor antagonist might activate hypothalamic insulin signaling, therefore enhancing hypothalamic insulin sensitivity. 

## Discussion

In our present study, the central effect of ghrelin receptor antagonists on NAFLD was explored. Beheshti *et al*. reported that different doses of [D-Lys-3]-GHRP-6 (0.2, 2, 20, and 80 n, 5µl; i.c.v) were delivered to rats, and the doses of the antagonist further than 2 nM saturated the ghrelin receptors (26). Sibilia *et al*. observed an antagonism of 3 nmol of [D-Lys-3]-GHRP-6 to 1 nmol exogenous of ghrelin (27). In comprehensive consideration of these results, we set the dose of [D-Lys-3]-GHRP-6 at 1 nmol to antagonize the endogenous ghrelin. The results revealed that ICV injection of [D-Lys-3]-GHRP-6 significantly reduced serum lipids, transaminase, and liver index, improved liver injury, and hepatocytes steatosis. Moreover, ICV injection of [D-Lys-3]-GHRP-6 significantly up-regulated the phosphorylation of hypothalamic PI3K/Akt/mTOR signaling proteins, indicating a significant improvement in hypothalamic insulin resistance. 

With the worldwide prevalence and mortality of NAFLD increasing (28, 29), it is an urgent need to develop effective strategies for prevention, surveillance, and intervention of NAFLD. Ghrelin is a key factor that links the central nervous system with peripheral tissues to regulate lipid metabolism and energy balance (30-32), In the NAFLD rats, we revealed that plasma AG levels increased slightly with no significant (18). However, there showed a significant up-regulation of AG and GHSR-1a expressions in the hypothalamus which exhibited positive correlations with HOMA-IR and hepatic TGs. Our results suggested that AG might induce insulin resistance and promote liver lipid accumulation via a central mechanism involved in the hypothalamus. Therefore, in the present study, we observed the effect of central administration of specific ghrelin receptor antagonist [D-Lys-3]-GHRP-6 on NAFLD. The results revealed that ICV injection of [D-Lys-3]-GHRP-6 in the NAFLD rats significantly decreased serum lipids, aminotransferase, and hepatic TGs levels. Morphological results suggested that ICV injection of [D-Lys-3]-GHRP-6 significantly inhibited hepatic steatosis and lipid deposits induced by HFD. Furthermore, HOMA-IR decreased after ICV injection of [D-Lys-3]-GHRP-6, indicating that insulin resistance was improved significantly. However, the intracranial route for administration of [D-Lys-3]-GHRP-6 cannot be translated to clinical settings. Fortunately, significant progress has been made in brain-targeted drug delivery in recent years. Non-invasive strategies such as intranasal delivery, P-glycoprotein inhibition, pro-drugs, and carrier/targeted nanocarrier-based delivery systems could be employed alone or often in combination for brain targeting (33). The present result inspires us to conduct more experiments to reveal the effect of central [D-Lys-3]-GHRP-6. Once the pre-clinical outcome seems promising, further research is warranted to explore drug delivery to the brain. Nose-to-brain nanoparticle drug delivery might be a candidate for [D-Lys-3]-GHRP-6 delivery to the brain (34, 35). Moreover, all experiments on [D-Lys-3]-GHRP-6 were conducted in animals and cells. It is difficult to predict the clinical safety of [D-Lys-3]-GHRP-6. As the research continues to deepen, we are looking forward to conducting clinical trials in the future.

The “Multiple hits” hypothesis was proposed in 2010 which claimed that many factors acted with each other to cause the pathogenesis of NAFLD, among which insulin resistance is an important one (36). As reported, HFD induced insulin resistance in our present study. Under physiological circumstances, insulin regulates hepatic glucose production through controlling lipolysis in the adipose tissues, therefore decreasing fatty acid influx to the liver. However, once insulin resistance occurred in NAFLD, the increased lipolysis in the adipose tissues resulted in abnormally increased glucose production in the liver, which further promotes hepatic *de novo* lipogenesis (37). Growing evidence suggests that the brain plays a vital role in regulating systemic metabolism and lipid balance. The signals of the body’s nutritional status are constantly being transduced to the brain regions responsive for energy-sensing and assessing. Meanwhile, the brain is also an insulin-sensitive organ and brain insulin has been identified as a crucial contributor to the regulation of food intake and energy balance (38). The insulin receptor is widely distributed in the hypothalamus. Insulin binds to the insulin receptor and activates the PI3K/Akt signaling pathway in the hypothalamus and then activates the downstream protein mTOR (39). In our present study, protein expression levels of p-PI3K, p-Akt, and p-mTOR in the hypothalamus decreased significantly in the NAFLD rats. The results suggested that hypothalamic insulin signaling was destroyed by HFD. After ICV injection of [D-Lys-3]-GHRP-6, the expression of the above proteins increased significantly. Blockade of central ghrelin receptor resulted in the improvement of insulin signaling which was beneficial to alleviate the hepatic lipid accumulation. Taken together, ICV injection of [D-Lys-3]-GHRP-6 ameliorates NAFLD via improving hypothalamic insulin resistance through the PI3K/Akt/mTOR pathway.

N=10; *P<0.05, **P<0.01, compared with the Control; ^#^*P<*0.05, ^##^*P<*0.01 compared with the NAFLD

ICV: Intracerebroventricular; NAFLD: Non-alcoholic fatty liver disease

N=10; *P<0.05, **P<0.01, compared with the Control; ^#^*P<*0.05, ^##^*P<*0.01, compared with the NAFLD

ICV: Intracerebroventricular; NAFLD: Non-alcoholic fatty liver disease

N=10; *P<0.01, compared with the Control; ^#^*P<*0.05, compared with the NAFLD

ICV: Intracerebroventricular; NAFLD: Non-alcoholic fatty liver disease; HOMA-IR: Homeostatic model assessment insulin resistance

*P<0.05, **P<0.01, compared with the Control; ^#^*P<*0.05, ^##^*P<*0.01, compared with NAFLD

NAFLD: Non-alcoholic fatty liver disease

## Conclusion

ICV injection of [D-Lys-3]-GHRP-6 significantly lowered serum lipids and transaminase and inhibited hepatic lipid accumulation and injury in the NAFLD rats. Furthermore, insulin resistance was improved and the phosphorylation of hypothalamic PI3K/Akt/mTOR proteins involved in insulin signaling was up-regulated. In a word, blockade of central ghrelin receptor can treat NAFLD possibly via the hypothalamic PI3K/Akt/mTOR signaling pathway to improve insulin resistance. 

## Authors’ Contributions

YG, YG,YJ and ZhX study conception and design; HZh data analyzing and draft manuscript preparation; HW and YG critical revision of the paper; HW and YG supervision of the research; YG Final approval of the version to be published. 

## Conflicts of Interest

None of the authors has personal or financial conflicts of interest. 
